# Normalization with genes encoding ribosomal proteins but not GAPDH provides an accurate quantification of gene expressions in neuronal differentiation of PC12 cells

**DOI:** 10.1186/1471-2164-11-75

**Published:** 2010-01-29

**Authors:** Lihan Zhou, Qing-En Lim, Guoqiang Wan, Heng-Phon Too

**Affiliations:** 1 Department of Biochemistry, National University of Singapore, 119260, Singapore; 2 Chemical Pharmaceutical Engineering, Singapore-Massachusetts Institute of Technology Alliance, 117576, Singapore

## Abstract

**Background:**

Gene regulation at transcript level can provide a good indication of the complex signaling mechanisms underlying physiological and pathological processes. Transcriptomic methods such as microarray and quantitative real-time PCR require stable reference genes for accurate normalization of gene expression. Some but not all studies have shown that housekeeping genes (HGKs), β-actin (ACTB) and glyceraldehyde-3-phosphate dehydrogenase (GAPDH), which are routinely used for normalization, may vary significantly depending on the cell/tissue type and experimental conditions. It is currently unclear if these genes are stably expressed in cells undergoing drastic morphological changes during neuronal differentiation. Recent meta-analysis of microarray datasets showed that some but not all of the ribosomal protein genes are stably expressed. To test the hypothesis that some ribosomal protein genes can serve as reference genes for neuronal differentiation, a genome-wide analysis was performed and putative reference genes were identified based on stability of expressions. The stabilities of these potential reference genes were then analyzed by reverse transcription quantitative real-time PCR in six differentiation conditions.

**Results:**

Twenty stably expressed genes, including thirteen ribosomal protein genes, were selected from microarray analysis of the gene expression profiles of GDNF and NGF induced differentiation of PC12 cells. The expression levels of these candidate genes as well as ACTB and GAPDH were further analyzed by reverse transcription quantitative real-time PCR in PC12 cells differentiated with a variety of stimuli including NGF, GDNF, Forskolin, KCl and ROCK inhibitor, Y27632. The performances of these candidate genes as stable reference genes were evaluated with two independent statistical approaches, geNorm and NormFinder.

**Conclusions:**

The ribosomal protein genes, RPL19 and RPL29, were identified as suitable reference genes during neuronal differentiation of PC12 cells, regardless of the type of differentiation conditions. The combination of these two novel reference genes, but not the commonly used HKG, GAPDH, allows robust and accurate normalization of differentially expressed genes during PC12 differentiation.

## Background

During development, neurons make networks of connections with other neurons by growing axons and dendrites. These neuronal out-growths are regulated by extracellular cues that signal to cells resulting in phenotypic changes. A major challenge is the identification of molecular mechanisms underlying this highly complex and interactive network in terms of the functions of genes and proteins[1].

Currently, transcriptomic methods are widely used as an initial step in unraveling the complex signaling mechanisms underlying physiological and pathological processes and in neuronal differentiation [2-5]. Gene microarray offers a high throughput platform for the analysis of the entire transcriptome to identify differentially expressed genes. Reverse transcription quantitative real-time PCR (RT-qPCR), with a wider dynamic range of quantification and higher assay sensitivity and precision, is often used to corroborate microarray findings [6,7]. Regardless of the method used, normalization, a critical process of adjusting the expression measurements between samples to compensate for various sources of variability in the assay, is essential to allow accurate comparisons of the results between different samples and conditions [8,9]. Normalization with internal reference gene is used to control for technical and biological variations introduced during both sample preparation and detection by RT-qPCR [10]. It has also been shown to be suitable for the normalization of partially degraded RNA samples [11-13].

With nearly all normalization methods, the assumption that one or more reference genes are constitutively expressed at near-constant levels under all experimental conditions is implicit and the expression levels of all other genes in the sample are then scaled to these reference genes accordingly. It is common to use reference genes selected from an assumed list of "housekeeping" genes (HKGs) which typically include transcripts such as GAPDH and ACTB [9,10,14]. A number of studies have now shown that the expressions of these genes, in some but *not all *experimental conditions, are altered significantly[15-18], thus, making the choice of using these HKGs for normalization uncertain without *a priori *knowledge.

A variety of approaches have been employed to enable better selection of reference genes. One approach is the use of statistical algorithms, for example, geNorm [14], Best keeper [19], NormFinder [20], Global Pattern Recognition [21], and Equivalence tests [22], to evaluate the relative expression stabilities of genes from a pool of predefined lists of candidates. While this approach is certainly more robust than using the single gene methods, it too is based on potentially unfounded assumptions about which genes may be stably expressed in the conditions studied. These genes are still required to be pre-selected and incorporated into the experimental designs without any *a priori *evidence to support their use. An alternative and less biased approach is the meta-analysis of large scale gene expression profiles to identify stably expressed genes [23-26]. A selected number of potential references genes can then be validated experimentally and the stability of expressions analyzed by the above mentioned statistical algorithms in defined experimental settings.

To date, reference genes validated for neuronal differentiation studies have not been reported yet. The present study aims to identify suitable reference genes during chemically induced neuronal differentiation of PC12, a cell-line derived from a pheochromocytoma of the rat adrenal medulla. Because of its unique cellular properties, suitability for genetic and biochemical manipulations, the PC12 cell-line is widely regarded as a convenient alternative to endogenous neuronal cells, and serves as a commonly used model system for studies on neuronal differentiation [27-29]. For example, in response to NGF, PC12 cells stop dividing, elaborate neuronal processes, are electrically excitable, and have the potential to form synapses when co-cultured with muscle cells [30]. Here, we measured the temporal expression of twenty novel candidate reference genes identified from microarray studies and the commonly used HKGs, ACTB and GAPDH, at various stages of PC12 differentiation. Based on two independent statistical approaches ["pairwise comparison" [14] and "model based variation" [20], the expressions of ribosomal protein genes RPL19 and RPL29 were found to be highly stable regardless of pharmacological treatments and stages of differentiation. The combination of the two reference genes was sufficient to allow robust and accurate normalization of differentiation related genes.

## Results

### Selection of candidate reference genes from microarray data

It has been suggested that suitable reference genes should be expressed in all experimental conditions and exhibit low coefficient of variation (CV) in their expressions [25,26,31,32]. In order to identify such reference genes, we first analyzed the expression profiles of 21,910 genes in naïve PC12 cells and those treated with NGF or GDNF and found 8,568 genes to be expressed in all conditions (detection p values < 0.05). We then analyzed the top 100 genes with the lowest CV (0.8% -1.45%) with two well accepted but different statistical approaches, "pairwise comparison" (geNorm) and "model based variation analysis" (NormFinder). The "pairwise comparison" approach assumes that a perfect pair of reference genes has a constant ratio across all experimental conditions. As such, geNorm evaluates the inter-conditional variability of the ratio between each pair of reference genes and calculates a gene stability measure M for each candidate [14]. However, with this method, tightly co-regulated genes will appear to be stable. The second algorithm, NormFinder, was employed to safeguard against such a pitfall of misidentifying expression invariant reference genes. This model-based variance estimation approach entails analysis of sample subgroups and calculates the variation of each candidate gene individually, based on both intra- and inter-group variation [20]. While geNorm measures relative stability, NormFinder measures absolute stability by decomposing the variance to biological and technical elements. With this method, the expressions of co-regulated genes can be distinguished. Despite the differences in algorithms and assumptions, both statistical methods were in agreement on the identity of the twenty most stable genes (Table 1), most of which are novel for the purpose of normalization studies. Interestingly, thirteen of these twenty candidate reference genes were ribosomal protein genes.

**Table 1:** Selection of candidate reference genes from microarray data

Gene symbol	Definition	Mean	geNorm	NormFinder
**RPL29**	Ribosomal protein L29	14.04	1	1
**RPL10a**	Ribosomal protein L10A	12.49	2	2
LOC292640	Vps20-associated 1 homolog	10.87	3	3
**LOC498143**	Similar to ribosomal protein L15	13.71	4	4
**LOC317275**	Similar to ribosomal protein L7-like 1	11.88	7	5
**RPS15**	Ribosomal protein S15	12.97	5	6
**ARBP**	Acidic ribosomal phosphoprotein P0	14.27	6	7
**RPL14**	Ribosomal protein L14	13.89	9	8
EEF1A1	Eukaryotic translation elongation factor 1 alpha 1	14.17	8	9
**RPS15A**	Ribosomal protein S15a	13.93	10	10
**RPL18**	Ribosomal protein L18	13.58	11	11
REPS1 (P)	RalBP1 associated Eps domain containing protein (predicted)	10.73	12	12
LOC363720	chromatin modifying protein 2B	10.61	14	13
CNOT8	CCR4-NOT transcription complex, subunit 8	11.00	15	14
RTCD1	RNA terminal phosphate cyclase domain 1	10.48	17	15
**RPL19**	Ribosomal protein L19	13.74	13	16
NDUFB6 (P)	NADH dehydrogenase (ubiquinone) 1 beta subcomplex, 6,	10.43	16	17
**RPL9**	Ribosomal protein L9	13.74	18	18
**LOC499803**	Similar to 40S ribosomal protein S3	13.76	19	19
**RPL3**	Ribosomal protein L3	14.03	20	20

ACTB	Actin, beta	13.85		
GAPDH	Glyceraldehyde-3-phosphate dehydrogenase	12.88		

Microarray analysis of the expression profiles of 21,910 genes in naïve PC12 cells and those treated with NGF or GDNF for 0.5 h and 72 h. Twenty candidate reference genes were selected based on pairwise comparison (geNorm) and model based variation (NormFinder) analysis of the top 100 genes with the lowest CV. The log2 transformed values of the average signal intensities among the 24 arrays were shown as Mean. Thirteen of the twenty genes were Ribosomal Protein Genes (Bold). Both ACTB and GAPDH were included for comparison but were not among the top 100 genes recommended by either statistical analysis.

### Real-time PCR validation of novel candidate reference genes

**Figure 1:**
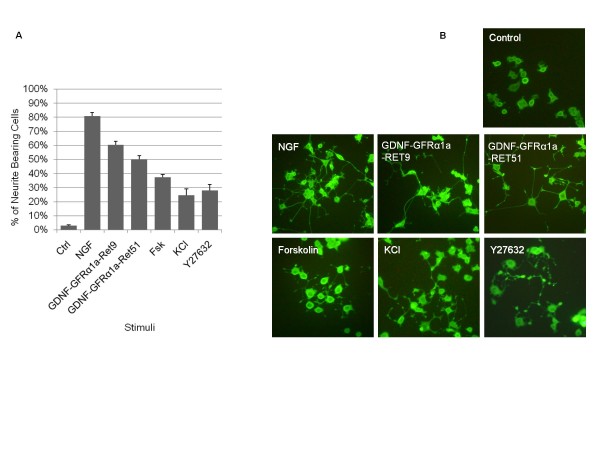
**Neuronal differentiation of PC12 cells**. **A**. Quantification of the percentage of PC12 cells bearing neurite of at least one cell body length, after 72 h of treatment with NGF (50 ng/ml), GDNF (50 ng/ml), Forskolin (10 μM), KCl (5 mM) and ROCK inhibitor Y27632 (25 μM). GDNF treatment was applied to PC12 cells stably expressing GDNF Family Receptor alpha 1a (GFRa1a) and co-receptor RET (either RET9 or RET51 isoforms), which were not endogenously expressed at detectable level in PC12 cells. All other stimulations were applied to wild type PC12 cells. **B**. Representative images of control and treated PC12 cells immuno-stained with anti-Neurofilament 200 antibody.

As one of the most extensively studied models for neuronal differentiation, PC12 cells respond to a broad spectrum of pharmacological agents, which trigger a myriad of intracellular signaling pathways leading to neuronal differentiation. In order to verify the general utility of the 20 selected putative reference genes (Table 1) in a broader range of experimental conditions, we differentiated PC12 cells with other stimuli (Forskolin [33], KCl [34] and ROCK inhibitor Y27632 [35]) in addition to NGF and GDNF. GDNF was applied to PC12 cells stably expressing GDNF Family Receptor alpha 1a (GFRa1a) and co-receptor RET (either RET9 or RET51 isoforms), which are not endogenously expressed at detectable levels in PC12 cells (data not shown). The percentage of PC12 cells differentiated by the five chemical stimuli was quantified (Figure 1A) and the axon-like features of the extended neurite were confirmed by immunocytochemical analysis with anti-Neurofilament-200 antibody (Figure 1B). The extent of neurite outgrowth was highly dependent on the stimuli used. NGF and GDNF stimulation induced longer neurite outgrowths than Forskolin, KCl or Y27632. Total RNA was collected at 0.5 h, 6 h, 24 h and 72 h from control and treated cells for each stimuli, with biological triplicates, that totaled 120 samples.

**Figure 2:**
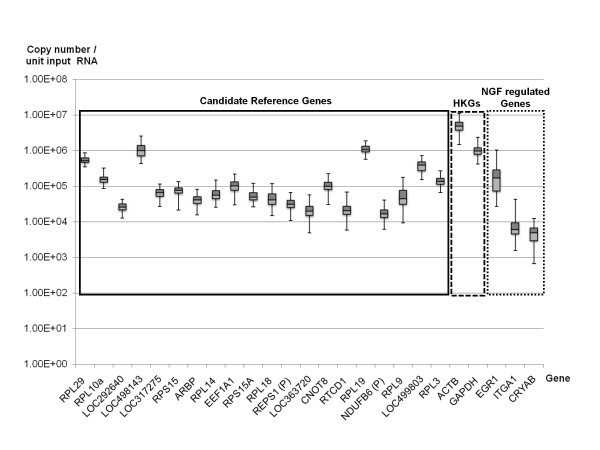
**Distribution of the expression levels of genes examined**. Box plot representation of the expression levels of twenty candidate reference genes (solid line), two housekeeping genes (dashed line) and three target genes (dotted line) among the 120 biological samples. The expression level of each gene was represented as the absolute copy number per unit input total RNA (0.0625 μg), quantified by RT-qPCR using linearized plasmid standards. Primer design, assay efficiency and intra- and inter-assay variations were reported in supplementary data (Additional File 1).

We analyzed the expression levels of the aforementioned twenty candidate reference genes, two most commonly used HKGs (GAPDH and ACTB), and three genes which are well known to be regulated by NGF using RT-qPCR (Figure 2). The expression levels of the twenty candidate reference genes and the two HKGs span three orders of magnitude. These reference genes were expressed at comparable levels or lower than the HKGs examined. For accurate determination of inter-assay variations and primer efficiencies, flanking regions of the genes (~300 bp) were amplified by PCR, sub-cloned and the sequences verified. These recombinant plasmids were then linearized and served as templates to construct standard curves. All the qPCR assays showed high efficiency of amplification (>90%) and low intra- and inter-assay variations (Additional File 1). All RNA samples showed RQI values of greater than 9, indicative of high quality and integrity (data not shown).

### Stabilities of candidate reference genes and common housekeeping genes

**Figure 3:**
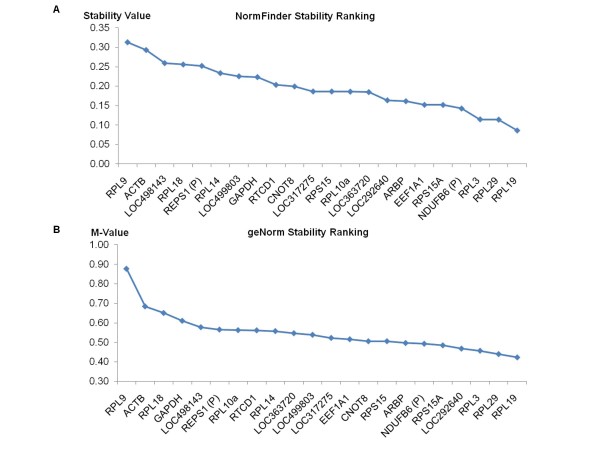
**Stability analysis of candidate reference genes and housekeeping genes**. Stability rankings of the twenty candidate reference genes and two most commonly used housekeeping genes ACTB and GAPDH, among all 120 biological samples, by NormFinder **(A) **and geNorm **(B)**. A low 'Stability Value' or 'M-value' correlates to higher gene expression stability.

Using both geNorm and NormFinder, we analyzed the expression stabilities of the twenty candidate reference genes and the two commonly used HKGs across all six differentiation conditions. Both statistical approaches recommended the same three ribosomal protein genes RPL19, RPL29 and RPL3 as the overall best reference genes (Figure 3). Pairwise variation analysis by geNorm showed that the combination of RPL19 and RPL29 is sufficiently stable (V2/3 = 0.107, less than the recommended cut-off of 0.15), thus excluding the need to incorporate a third reference gene RPL3 for normalization of target gene expression. Notably, neither GAPDH nor ACTB were recommended.

Further analysis of candidate gene stabilities in each treatment group (Additional File 2A) or at specific time point (Additional File 2B) revealed that the stability rankings of candidate genes do vary among different subgroups. However, with the exception of RPL29 in KCl treated samples, the two genes RPL19 and RPL29 were consistently ranked among top 5 in all subgroups. In contrast, the stability rankings of GAPDH and ACTB varied considerably among different subgroups and they were ranked among the least stable ones within the group of 22 genes in several subgroups. The data indicated that the two novel candidate genes RPL19 and RPL29 have higher expression stabilities than both GAPDH and ACTB, and may serve as better normalizers for gene expression in neuronal differentiation of PC12 cells.

### Comparison of the normalization factors generated by different reference gene(s)

**Figure 4:**
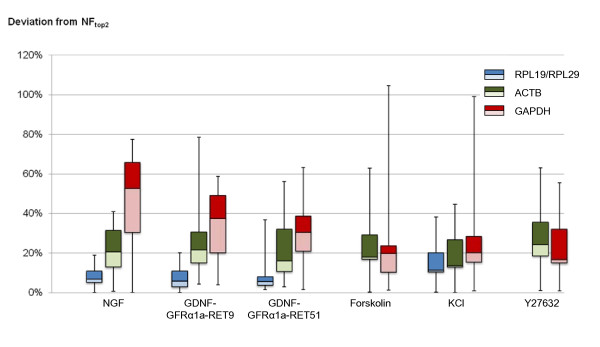
**Comparison of the normalization factors calculated using different reference gene(s)**. Normalization factors (NFs) calculated with RPL19/RPL29, ACTB and GAPDH were compared to that calculated by the top 2 reference genes (NF_top2_) as recommended by both NormFinder and geNorm, for each stimulus. The percentage deviations of NF_RPL19/RPL29_; NF_ACTB_; NF_GAPDH _from NF_top2 _(|NF_x_-NF_top2_|/NF_top2_) were represented by box plot. The 25^th ^percentile to the 75^th ^percentile (boxes), and ranges (whiskers) were shown.

To account for possible variations introduced during sample preparation and measurements, raw expression profiles of target genes were scaled by a normalization factor (NF) calculated based on independent measurement of one or more internal reference genes. The variation between NFs generated by different reference genes is thus directly reflective of the variation in the final target gene expression values normalized by different reference genes. We noticed that although RPL19 and RPL29 were ranked as the overall best pair of reference genes, they were not necessarily the best pair for each treatment subgroup. To test the robustness of these two genes across different treatments, we compared the normalization factors calculated based on RPL19 and RPL29 (NF_RPL19/RPL29_) to that of the most stable pair of reference genes (NF_top2_) in each treatment subgroup. Similarly, we examined the differences between NF_top2 _and NFs calculated based on the commonly used HKGs, ACTB (NF_ACTB_) or GAPDH (NF_GAPDH_). The deviations of each NF from NF_top2 _are represented in Figure 4 (see Additional File 3 for details of calculation). The NF_RPL19/RPL29 _values were found to least deviate from NF_top2 _in NGF, GDNF and KCl subgroups, and had zero deviation in Fsk and Y27632 subgroups as RPL19 and RPL29 were ranked top 2. In contrast, NF_ACTB _and NF_GAPDH _differed substantially from NF_top2 _in many instances, reflective of their varying stabilities across different treatments.

### Effect of different reference genes on the interpretation of target gene regulation

**Figure 5:**
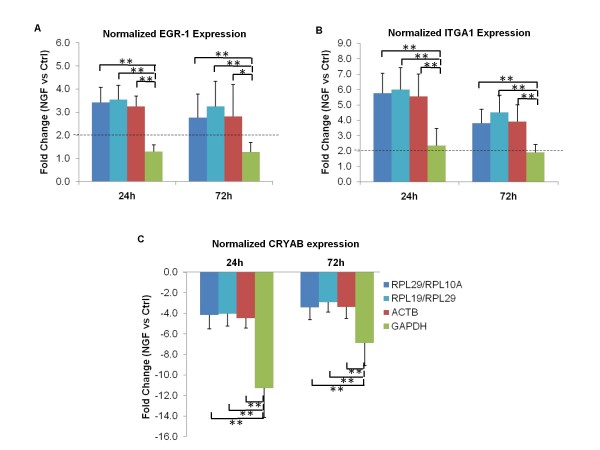
**Fold changes in target gene expressions normalized using different reference gene(s)**. Fold changes in transcript expressions of Egr-1 **(A)**, Integrin alpha 1, ITGA1 **(B)**, Crystallin alpha b, CRYAB **(C)**, in NGF treated samples relative to that of control were normalized by (1) geometric mean of RPL10a/RPL29; (2) geometric mean of RPL19/RPL29; (3) ACTB or (4) GAPDH. Normalization by GAPDH led to significant quantitative underestimations of EGR1 and ITGA1 upregulation and overestimation of CRYAB downregulation. Dotted line represents the 2-fold difference between treatment and control subjects, a cut off commonly used to distinguish significant changes from insignificant ones. Significant differences between fold changes normalized by various reference gene(s) were calculated using the paired Student's *t-test*. A value of p < 0.05 was considered significant (**p < 0.01; *p < 0.05).

Next, the possibility that using scaling factors of NF_RPL19/29_, NF_ACTB _or NF_GAPDH _may substantially alter the interpretation of target gene expression regulation in NGF induced neuronal differentiation was investigated. The relative fold changes of EGR1, ITGA1 and CRYAB expressions normalized by the three NFs were compared to the values normalized by NF of the top 2 genes (NF_RPL29/RPL10A_). No statistically significant differences were observed among NF_RPL29/RPL10A_, NF_RPL19/RPL29_, and NF_ACTB _normalized values; whereas NF_GAPDH _normalized fold changes were significantly different (Figure 5A-C). In the case of EGR1 and ITGA1, the use of GAPDH as reference gene resulted in the underestimation of target genes expressions, leading to false negative conclusions when a two-fold cut off was applied (Figure 5A-B). On the other hand, normalization by GAPDH resulted in the significant over-estimation of the down-regulation of CRYAB in NGF treated samples (Figure 5C).

**Figure 6:**
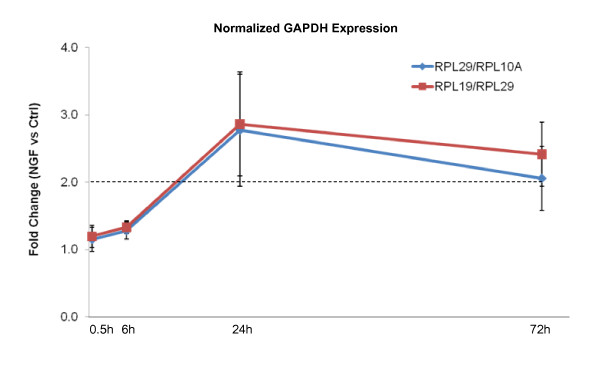
**Upregulation of GAPDH transcript expression in NGF induced neuronal differentiation**. Fold changes in transcript expression of GAPDH in NGF treated samples relative to that of control were normalized by (1) geometric mean of RPL10a/RPL29; or (2) geometric mean of RPL19/RPL29. Dotted line represents the 2-fold difference between treatment and control cells.

The clearly different expression profiles of EGR1, ITGA1, and CRYAB when normalized to GAPDH raised the possibility that GAPDH expression could be regulated over the course of NGF induced differentiation. Normalization of GAPDH expression by the NF of the top 2 genes (NF_RPL29/RPL10A_) and the NF_RPL19/RPL29_, revealed that GAPDH expression was indeed significantly elevated (>2.5 fold at 24 h) in NGF-stimulated PC12 cells (Figure 6). A more detailed analysis of the kinetics of GAPDH expression over time revealed that expression of GAPDH indeed increased over a period of 28 h (Additional File 4). As a result, the use of GAPDH as a single, unverified reference gene would invariably lead to erroneous interpretation of target gene regulation.

Similarly, we investigated the effect of different reference gene(s) on normalized target gene expressions in GDNF, Forskolin, KCl and ROCK inhibitor Y27632 treated samples. Similar to the case of NGF treatment, with GDNF stimulated PC12-GFRa1a/RET9 and PC12-GFRa1a/RET51 cells, normalization by GAPDH resulted in the underestimations of GDNF induced upregulation of EGR1 and ITGA1 expressions; and over-estimated CRYAB down-regulation (Additional File 5A, B). Interestingly, normalization by ACTB was found to overestimate the expression of EGR1 and ITGA1 expressions in PC12-GFRa1a/RET51 but not RET9 cells (Additional File 5B, 72 h), highlighting the subtle differences between GFRa1a/RET9 and GFRa1a/RET51 systems.

For Forskolin and ROCK inhibitor Y23672 differentiated samples, normalization by ACTB consistently led to the over-estimations of target gene expressions (Additional File 5C, D). Depending on the time point analyzed, normalization by GAPDH was shown to result in either underestimations or overestimations of target gene expressions (Additional File 5C, D). In KCl treated samples, no statistical significant difference was observed among NF_top2 (RPL19/REPS1)_, NF_RPL19/RPL29_, NF_ACTB _and NF_GAPDH _normalized target gene expression, which suggested that all four were acceptable reference gene(s) for this particular experimental condition (data not shown).

It is thus evident that the stabilities of the two most commonly used HKGs, GAPDH and ACTB vary across different experimental conditions during neuronal differentiation of PC12 cells. They were acceptable reference genes under some conditions but may significantly under- or over-estimate target gene expression under others. On the contrary, the two novel candidate reference genes RPL19 and RPL29 were stably expressed among all conditions analyzed and allowed accurate normalization of differentially regulated genes during PC12 differentiation. It is worthy to note that at early time points (0.5 h and 6 h), the expressions of EGR1, ITGA1 and CRYAB did not show any significant differences when scaled with either NF_top2, _NF_RPL19/RPL29_, NF_ACTB _or NF_GAPDH _(data not shown). This is consistent with the observation that the expression of GAPDH did not change significantly at the early time points (Figure 6. and Additional File 4).

## Discussion

Twenty candidate reference genes that showed little variation but high expression in PC12 cells differentiated with NGF and GDNF were first selected from microarray datasets using two independent statistical algorithms. Together with two well studied HKGs, the expression stabilities of these candidate reference genes were further analyzed using RT-qPCR in cells differentiated with other stimuli. From these studies, unexpectedly, RPL19 and RPL29 but not the HKGs, were identified as suitable reference genes that can be used for normalization of gene expression in neuronal differentiation of PC12 induced by a variety of chemical stimuli.

Neuronal differentiation is a process where cells undergo enormous morphological changes, over a period of several days. It is accompanied by substantial biochemical changes including cell cycle exit [36], changes in metabolism [37,38] and alteration in structural proteins [39,40]. Since the commonly used reference genes are mostly structural proteins or enzymes involved in metabolism, it is especially important to validate the stabilities of these genes during the process of differentiation. Many of these studies investigated gene expression changes in PC12 but few have evaluated the suitability of HKGs as normalizers in this model. Our microarray analysis revealed that a group of novel candidate genes was more stably expressed than commonly used HKGs ACTB and GAPDH, suggesting that ACTB and GAPDH may not be ideal reference genes in neuronal differentiation of PC12 cells.

In an effort to gain an insight into the temporal regulation of genes during neuronal differentiation, it is necessary that the reference genes used are stably expressed over a period of days. GAPDH and ACTB have been used for normalization in more than 90% of previous reports [41], often without proper validation of their stabilities. Numerous publications have reported that such HKGs can be differentially expressed under various experimental paradigms and are therefore inappropriate for normalization [9,15,16]. However, there are also recent reports that these HKGs are stably expressed and can serve as reference genes [17,18,42]. Most genes, including GAPDH and ACTB, examined in this study were stable at early stages of differentiation (0.5 h or 6 h). However, as differentiation proceeded with dramatic morphological changes and concomitant biochemical changes, the instability of expressions of GAPDH and many of the genes examined was obvious. In the case of GAPDH, this instability issue correlated well with the temporal increase of expression level, which peaked at 28 h and was sustained over a period of 72 h. While GAPDH may still serve as a reference gene for PC12 cells under specific conditions, the validity of using this gene and other less stable ones should be experimentally verified. However, the two RP genes (RPL19 and RPL29) that showed good stability in expression over the period of differentiation provided an optimal pair of reference genes for the entire period of and various experimental conditions for neuronal differentiation.

Among the twenty candidate genes selected, thirteen were ribosomal protein genes, suggesting that the family of ribosomal protein genes may become yet another source of reference genes. Several recent publications have validated and recommended the use of ribosomal protein genes as reference genes [23,25], while others have reported their tissue-dependent variations [43]. A plausible explanation for such disparity is the large number of ribosomal protein genes present in mammalian systems (80 genes in human, mouse and rat genome), which may be stably or differentially expressed depending on the tissue type or experimental conditions. At present, relatively little is known about these mammalian ribosomal proteins, as compared to their bacterial and archael counterparts [44]. While bacterial ribosomal protein genes exist largely in clusters, the mammalian RP genes are dispersed throughout the genome [45]. Some have suggested that all of these proteins are intimately involved in ribosome production and could be co-regulated. Depletion of a particular ribosomal protein would generally cause a reduction of all other ribosomal proteins in the same ribosome sub-unit [46]. Other reports have shown that some ribosomal protein genes could be regulated independent of others [47]. Recently, extra-ribosomal functions of some of these proteins have been reported [48-51], suggesting that they may be individually regulated. A previous study comparing random ESTs from naïve and NGF-treated PC12 cells, reported an NGF-promoted decrease in the expressions of RPL19 [52]. However, this decrease in RPL19 was not observed in other studies using SAGE [53] or microarray [2]. Similar to the latter studies, we too did not observe changes in RPL19 transcripts with NGF-treated PC12. Moreover, the SAGE study but not the microarray analysis reported a significant decrease in RPL29 expression. Using both microarray and RT-qPCR, we have also shown that RPL29 was unchanged when the cells were differentiated. The reasons for these discrepancies are unclear and may be due to the differences in methods used. We have shown here by quantitative real-time PCR that some ribosomal protein genes, RPL19 and RPL29, are highly stably expressed and are thus suitable reference genes, whereas others like RPL9 and RPL18 can vary significantly during differentiation.

Unlike some studies that attempted to identify ideal reference genes through meta-analysis of many publically available microarray data which includes a diverse range of tissue types and experimental conditions, this study was designed to specifically identify a set of suitable reference genes for PC12 cells undergoing neuronal differentiation. We have performed both the microarray analysis and RT-qPCR validation on biological samples prepared with the same techniques and reagents, thus minimizing variations introduced by differences in sample preparation methods and assay platforms. We have also systematically evaluated the effect of the use of NFs of inappropriate reference gene(s) on the expression changes of the target genes and the erroneous results they resulted in. Thus, with neuronal differentiation of PC12 cells, scaling with the geometric means of the expressions of RPL19 and RPL29 is recommended for the accurate normalization of gene expression. Whether these two genes are suitable for normalization of neuronal differentiation in other systems remains to be evaluated.

## Conclusions

Twenty novel candidate reference genes were identified and their expression stabilities were analyzed and compared to that of commonly used HKGs ACTB and GAPDH. Through this systematic study that included both microarray analysis and RT-qPCR, we have found two ribosomal protein genes RPL19, and RPL29 to be stably expressed during neuronal differentiation of PC12 cells, induced by five different chemical stimuli, over 72 h. The combination of these two novel reference genes allowed robust and accurate normalization of differentially expressed genes, regardless of stimuli and stages of differentiation. In contrast, the use of an inappropriate reference gene like GAPDH led to significant erroneous estimation of differentially expressed genes.

## Methods

### Cell Culture

The rat pheochromocytoma cell line PC12 (catalog # CRL-1721; American Type Culture Collection) cells were grown in DMEM supplemented with 10% heat-inactivated fetal bovine serum (FBS; Hyclone, Logan, UT) and 5% Horse Serum (HS), in a humidified atmosphere with 5% CO_2 _at 37°C. Wild type PC12 cells, that do not endogenously express GFRa or RET (data not shown), were co-infected with murine GFRa1a (NM_010279) and RET9 (NM_001080780) or RET51 (NM_009050) in pQCXIN or pQCXIH vector by retro-viral infection (Clontech, Mountain View, CA) and selected with 0.4 mg/ml G418 and 0.1 mg/ml Hygromycin, over a period of 2 months.

### Differentiation, sample collection and assessment of neurite outgrowth

Two million wild type or infected PC12 cells were seeded in 75 cm^2 ^flask (NUNC, Finland) overnight, in DMEM supplemented with 10% FBS and 5% HS, followed by serum depletion for 12 h. PC12 cells were then treated with 50 ng/ml recombinant human GDNF (Peprotech, NJ), 50 ng NGF (Peprotech, NJ), 10 μM Forskolin (Sigma, St. Louis, MO), 5 mM KCl (Sigma, St. Louis, MO) or 25 μM ROCK inhibitor Y27632 (Calbiochem, USA) in DMEM to induce neurite outgrowth. Total RNA was isolated from control and treated cells at 0.5, 6, 24 or 72 h. For neurite outgrowth assessment, cells bearing at least one neurite with the length equivalent to the cell bodies were scored at 72 h by independent observers. More than 400 cells from three different fields were counted per flask.

### Immunocytochemistry

Control and treated PC12 Cells were fixed with 4% paraformaldehyde in 1xPBS for 15 min at 37°C, and subsequently in methanol at -20°C for an additional 15 min. After three washes with 1xPBS, cells were permeabilized and blocked with normal goat serum (1:10; Dako, Glostrup, Denmark) in 0.5% Triton X-100/PBS for 30 min at 37°C. The cells were then incubated with high-molecular-weight neurofilament protein (NF-200) antibody (Sigma, St. Louis, MO) at 1:80 dilution in 0.1% Triton X-100/0.1% BSA/1xPBS for 1 h at 37°C and washed three times in 1xPBS. Subsequently, the cells were incubated with goat anti-rabbit fluorescent secondary antibody (Alexa Fluor 488; Invitrogen, CA) diluted 1:1000 in 0.1% Triton X-100/0.1% BSA/1xPBS for 1 h. The cells were then washed three times in 1xPBS. Image acquisition was performed using the Zeiss inverted Axovert 25 microscope equipped with fluorescence detection (Oberkochen, Germany).

### RNA Purification and cDNA Preparation

Total RNA from PC12 cells was prepared using TRIzol reagent (Invitrogen, CA) according to manufacturer's instruction. Total RNA was collected from samples in quadruplicate at each treatment time point and the integrity of the RNA validated by denaturing agarose gel electrophoresis and using the StdSens analysis chip on the Experion Automated Electrophoresis System (BioRad, CA) according to manufacturer's instructions. The Experion Automated Electrophoresis System assigns a RQI to each RNA electropherogram which ranges from 10 (intact RNA) to 1 (completely degraded RNA). RNA concentration was quantified using a NanoDrop ND-1000 spectrophotometer (Thermo Scientific, Wilmington, DE), and the 260/280 and 260/230 ratios were examined for protein and solvent contamination. Five micrograms of total RNA were reverse transcribed in a total volume of 20 μl containing 400 U of ImpromII and 0.5 μg of random hexamer (Promega, Madison, WI) for 60 min at 42°C according to the manufacturer's instructions. The reaction was terminated by heating at 70°C for 5 min, and the cDNA was diluted 1:20 for quantitative real-time PCR.

### Microarray

PC12 cells were seeded on 25 cm^2 ^flask in complete medium and subsequently incubated for 12 h in serum free DMEM. The cells were then treated with GDNF (50 ng/ml) or NGF (50 ng/ml) for 0.5 h or 72 h in duplicates. Total RNA was isolated, quantified and integrity verified before it was amplified using Ambion Illumina RNA Amplification kit (Ambion, TX, USA). Briefly, total RNA (500 ng) was reverse transcribed by ArrayScript in the presence of T7 Oligo(dT) primer. Second strand of the cDNA was synthesized by DNA polymerase at 16°C for 2 h. The cDNA was purified and *in vitro *transcribed with T7 RNA polymerase and biotin-NTPs. Biotin-labeled cRNA samples were purified and quantified by ND-1000 spectrophotometer (NanoDrop, Fisher Thermo, DE, USA). Each cRNA (750 ng) was hybridized to RatRef-12 Expression BeadChip (Illumina, San Diego, CA, USA) containing 22,523 probes for a total of 21,910 rat genes selected primarily from the NCBI RefSeq database (Release 16) according to instruction provided by Experienced User Card (11286340 Rev A, Illumina). After hybridization, washing and blocking, the BeadChip was incubated with Streptavidin-Cy3 solution (Amersham Biosciences, Piscataway, NJ, USA). Fluorescent signals were obtained from scans on the high resolution Illumina BeadArray reader, using a two-channel, 0.8 μm resolution confocal laser scanner. The Illumina BeadStudio software (Version 2.0) was used to extract fluorescence intensities and the raw fluorescent data was background subtracted and used for analysis. Background is defined as the average signal intensity estimated from the negative control bead types. Outliers are removed using the median absolute deviation method. Detection p-values produced by the BeadStudio software were corrected for multiple hypothesis testing.

### Primer Design and Plasmid Standards

The Genbank accession for each candidate reference gene was retrieved from the Illumina microarray probe set and compared to the NCBI RefSeq database (Release 16; http://www.ncbi.nlm.nih.gov. Transcript splicing sites were retrieved from Ensembl http://www.ensembl.org. Where more than one transcript matched the probe, the sequences were aligned and the primers were designed to amplify the consensus region. Vector NTI Advance 10 was used to design two sets of primers for each target gene. The first set of primers generates an amplicon of ~300 bp and is used as a template for RT-qPCR of the targeted gene. The template was subcloned into pGEMT-easy (Promega) vector as previously described [54]. The second set of primers was used for RT-qPCR and was designed to amplify a ~100 bp region within the ~300 bp template. Both primer sets were exon spanning to avoid amplification from genomic sequences. Where possible, primers for RT-qPCR were designed to target the same exons used in the Illumina Expression BeadChip. All primer sequences were evaluated for possible false priming to known rat sequences using the NCBI BLAST tool http://blast.ncbi.nlm.nih.gov/. All products generated after amplifications were verified by gel-electrophoresis and DNA sequencing.

### Quantitative Real-Time PCR

Real-time PCR was performed on Biorad iCycler 4 Real-Time PCR Detection System (Bio-Rad, Hercules, CA) using SYBR Green I. The threshold cycles (Ct) were calculated using the iQ5 Optical system software version 2.0. Real-time PCR was performed after an initial denaturation for 10 min at 95°C, followed by 40 cycles of 30 s denaturation at 95°C, 30 s annealing at 60°C, and 30 s extension at 72°C. Fluorescent detection was performed at the annealing phase. Melt curve analysis was carried out at the end of the cycling to confirm that a single product had been amplified. Primer dimer formation in all the assays showed distinct melt characteristics from the correct amplicons. The reaction was performed in a total volume of 40 μl in 1× XtensaMix-SG (BioWORKS, Singapore), containing 2.5 mM MgCl_2_, 10 pmol of each primer, and 0.5 U of KlearTaq Hotstart DNA polymerase (KBioscience, UK). All real-time PCR quantification was performed simultaneously with linearized plasmid standards and a non-template control [54]. As PCR is an exponential process, it can be described by the equation, N_n _= N_0_(1 + ε)^n^, where N_n _is the number of target molecules at cycle n, N_0 _is the initial number of target molecules, ε is the efficiency of amplification and n is the number of cycles. The efficiency of target amplification of an assay was determined from the slope of a plot of Ct (Threshold cycle) versus -log_10 _concentration of the initial number of target molecules. High efficiency of amplification has a slope approaching the value of 3.32 cycles for every 10-fold dilution of the target. The gene expression levels were interpolated from standard curves and expressed as absolute copy numbers. All Real-time PCR experiments were compliant with the MIQE (Minimum Information for Publication of Quantitative Real-Time PCR Experiments) guidelines [55].

### Statistical data analysis

Gene expression stability analysis using two publicly available software tools, geNorm http://medgen.ugent.be/genorm/ and NormFinder http://www.mdl.dk/, were carried out according to authors' instruction.

## Authors' contributions

LZ carried out most of the sample preparation and real-time PCR, performed statistical analysis of the data, participated in the microarray analysis and drafted the manuscript. QEL designed the primers, performed the molecular cloning, and participated in sample preparation, real-time PCR and data analysis. GW performed the microarray analysis, participated in sample preparation and real-time PCR. HPT conceived of the study, and participated in its design, coordination, pipetting of samples and reviewed the manuscript. All authors read and approved the final manuscript.

## Supplementary Material

Additional file 1 **RT-qPCR assay design and performance**. Efficiencies of amplification and inter/intra-assay precisions of the assays used to measure the twenty candidate reference genes, two commonly used housekeeping genes and three target genes quantified in this study. Click here for file

Additional file 2 **Stability rankings of twenty candidate reference genes, ACTB and GAPDH in treatment and time-point subgroups**. Stability rankings were determined by *NormFinder (Italic) *and geNorm, for each stimulus (Additional File 2A) or time point (Additional File 2B) subgroup. The top two candidate genes (RPL19 and RPL29) in overall ranking (Figure 4) were bolded in red and the two HKGs were bolded and highlighted in grey.
Click here for file

Additional file 3 **Calculations of the deviation from NF_top2_**. Illustration of the calculation of the deviation of different normalization factors (NF_RPL19/RPL29_; NF_ACTB _and NF_GAPDH _) from NF_top2 _(NF_RPL10A/RPL29 _for NGF group), in 12 control and 12 NGF treated samples. Click here for file

Additional file 4 **Time course analysis of GAPDH expression in NGF induced PC12 differentiation**. A detailed time course analysis showing the up-regulation of GAPDH transcript expression by NGF treatment in PC12 cells, normalized by the geometric mean of RPL19 and RPL29. Click here for file

Additional file 5 **Normalized target gene expression regulation in PC12 cells differentiated with GDNF, Forskolin and Y27632**. Fold changes in transcript expressions of Egr-1 (**i**), Integrin alpha 1, ITGA1 **(ii)**, and Crystallin alpha b, CRYAB (**iii**), in GDNF-GFRa1a-RET9 (**A**), GDNF-GFRa1a-RET51 (**B**), Forskolin (**C**), Y27632 (**D**) treated samples relative to that of control were normalized by geometric mean of top 2 reference genes in each subgroup; geometric mean of RPL19/RPL29; ACTB or GAPDH. Normalization by ACTB resulted in the over-estimation of target gene expression. Normalization by GAPDH led to either under- or over-estimation of target gene expression. Dotted line represents the 2-fold difference between treatment and control subjects, a cut off commonly used to distinguish significant changes from insignificant ones. Significant differences between fold changes normalized by various reference gene(s) were calculated using the paired Student's *t test*. A value of p < 0.05 was considered significant (**p < 0.01; *p < 0.05) Click here for file
