# Supplementary material for: Normalization with genes encoding ribosomal proteins but not GAPDH provides an accurate quantification of gene expressions in neuronal differentiation of PC12 cells
Source: BMC Genomics. 2010 Jan 29;11:75. doi: 10.1186/1471-2164-11-75 (PMC2831847; doi:10.1186/1471-2164-11-75)
Supplement: Additional file 3 — Calculations of the deviation from NFtop2. Illustration of the calculation of the deviation of different normalization factors (NFRPL19/RPL29; NFACTB and NFGAPDH ) from NFtop2 (NFRPL10A/RPL29 for NGF group), in 12 control and 12 NGF treated samples. [file 1471-2164-11-75-S3.PDF]

Normalization factors

	Top2	RPL19/RPL29	GAPDH	ACTB
**Ctrl-0.5h-1**	0.662	0.706	0.186	0.396
**Ctrl-0.5h-2**	0.699	0.787	0.277	0.599
**Ctrl-0.5h-3**	0.820	0.883	0.275	0.707
**Ctrl-6h-1**	0.764	0.812	0.260	0.488
**Ctrl-6h-2**	0.796	0.879	0.272	0.789
**Ctrl-6h-3**	0.875	0.892	0.249	0.641
**Ctrl-24h-1**	0.862	0.941	0.350	0.721
**Ctrl-24h-2**	0.779	0.858	0.175	0.461
**Ctrl-24h-3**	0.698	0.811	0.204	0.595
**Ctrl-72h-1**	0.668	0.794	0.482	0.459
**Ctrl-72h-2**	0.825	0.882	0.363	0.641
**Ctrl-72h-3**	0.695	0.802	0.372	0.436
**NGF-0.5h1**	0.728	0.816	0.295	0.718
**NGF-0.5h-2**	0.773	0.741	0.286	0.631
**NGF-0.5h-3**	0.735	0.783	0.271	0.800
**NGF-6h-1**	0.839	0.855	0.332	0.845
**NGF-6h-2**	0.961	0.980	0.410	0.887
**NGF-6h-3**	0.939	0.956	0.384	1.000
**NGF-24h-1**	0.944	1.000	0.709	0.848
**NGF-24h-2**	0.959	0.975	0.659	0.716
**NGF-24h-3**	0.774	0.896	0.756	0.560
**NGF-72h-1**	0.857	0.793	0.935	0.580
**NGF-72h-2**	1.000	0.991	1.000	0.625
**NGF-72h-3**	0.709	0.685	0.889	0.538

Deviation from NF_{top2}

$|NF_x - NF_{top2}| / NF_{top2}$

	RPL19/RPL29	GAPDH	ACTB
**Ctrl-0.5h-1**	6.7%	71.8%	40.2%
**Ctrl-0.5h-2**	12.6%	60.3%	14.3%
**Ctrl-0.5h-3**	7.7%	66.5%	13.8%
**Ctrl-6h-1**	6.2%	66.0%	36.1%
**Ctrl-6h-2**	10.4%	65.9%	0.9%
**Ctrl-6h-3**	1.9%	71.5%	26.7%
**Ctrl-24h-1**	9.2%	59.4%	16.4%
**Ctrl-24h-2**	10.1%	77.5%	40.9%
**Ctrl-24h-3**	16.2%	70.8%	14.7%
**Ctrl-72h-1**	18.9%	27.9%	31.3%
**Ctrl-72h-2**	6.9%	56.0%	22.3%
**Ctrl-72h-3**	15.4%	46.5%	37.3%
**NGF-0.5h1**	12.1%	59.5%	1.3%
**NGF-0.5h-2**	4.1%	63.0%	18.3%
**NGF-0.5h-3**	6.5%	63.1%	8.8%
**NGF-6h-1**	1.9%	60.5%	0.7%
**NGF-6h-2**	1.9%	57.4%	7.8%
**NGF-6h-3**	1.9%	59.1%	6.5%
**NGF-24h-1**	5.9%	24.9%	10.2%
**NGF-24h-2**	1.6%	31.3%	25.4%
**NGF-24h-3**	15.8%	2.2%	27.6%
**NGF-72h-1**	7.5%	9.1%	32.3%
**NGF-72h-2**	0.9%	0.0%	37.5%
**NGF-72h-3**	3.5%	25.3%	24.1%

Box Plot

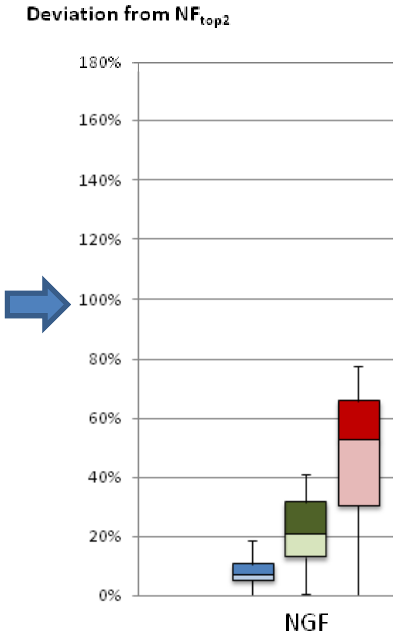

Additional file 3 – Calculations of the deviation from NF $_{top2}$.

Illustration of the calculation of the deviation of different normalization factors ($NF_{RPL19/RPL29}$; NF_{ACTB} and NF_{GAPDH}) from NF_{top2} ($NF_{RPL10A/RPL29}$ for NGF group), in 12 control and 12 NGF treated samples.
