# Supplementary material for: Normalization with genes encoding ribosomal proteins but not GAPDH provides an accurate quantification of gene expressions in neuronal differentiation of PC12 cells
Source: BMC Genomics. 2010 Jan 29;11:75. doi: 10.1186/1471-2164-11-75 (PMC2831847; doi:10.1186/1471-2164-11-75)
Supplement: Additional file 4 — Time course analysis of GAPDH expression in NGF induced PC12 differentiation. A detailed time course analysis showing the up-regulation of GAPDH transcript expression by NGF treatment in PC12 cells, normalized by the geometric mean of RPL19 and RPL29. [file 1471-2164-11-75-S4.PDF]

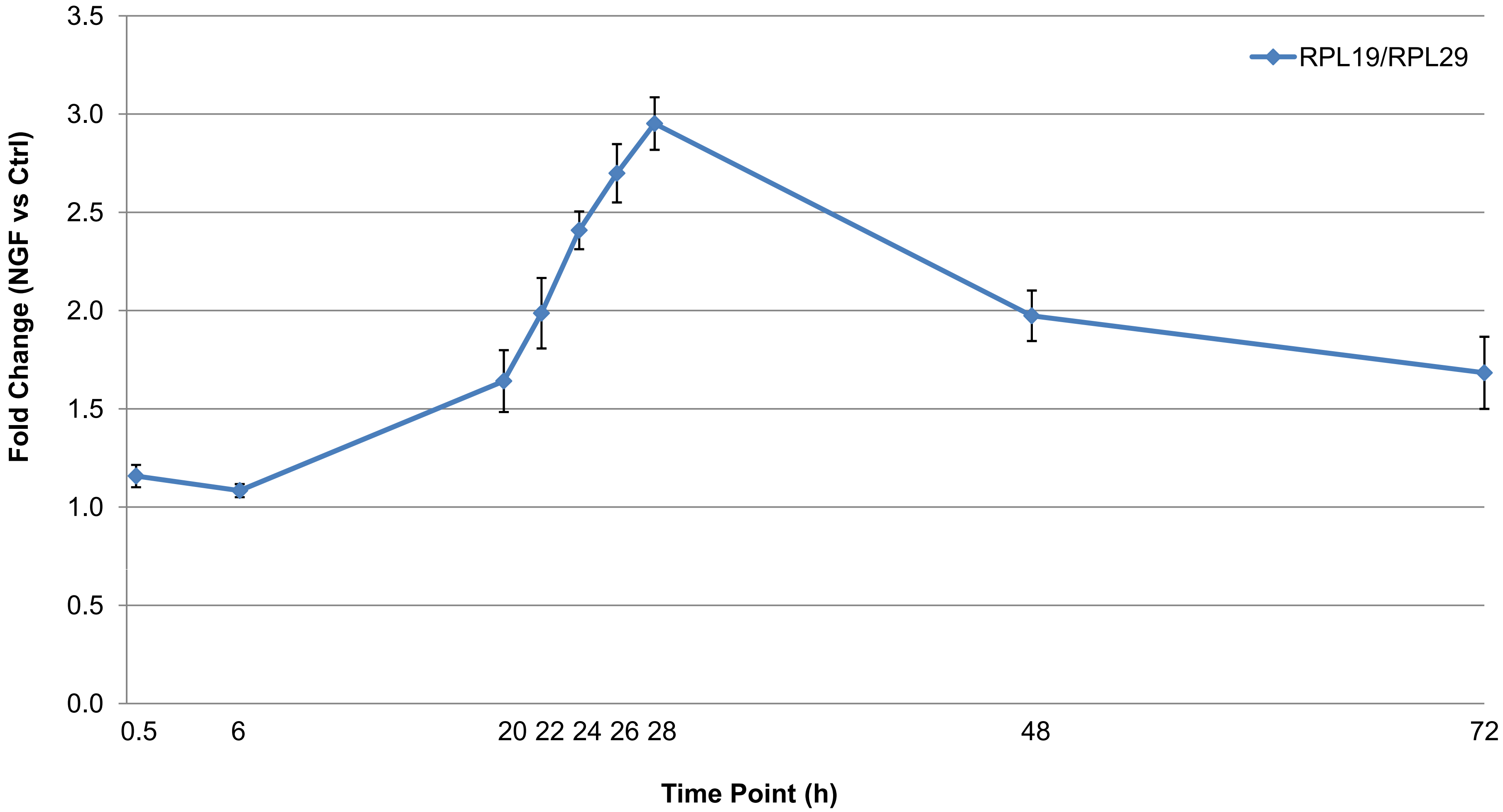

Additional file 4 – Time course analysis of GAPDH expression in NGF induced PC12 differentiation

A detailed time course analysis showing the up-regulation of GAPDH transcript expression by NGF treatment in PC12 cells, normalized by the geometric mean of RPL19 and RPL29.
