# Supplementary material for: Normalization with genes encoding ribosomal proteins but not GAPDH provides an accurate quantification of gene expressions in neuronal differentiation of PC12 cells
Source: BMC Genomics. 2010 Jan 29;11:75. doi: 10.1186/1471-2164-11-75 (PMC2831847; doi:10.1186/1471-2164-11-75)

### Additional file 5 – Normalized target gene expression regulation in PC12 cells differentiated with GDNF, Forskolin and Y27632.

Fold changes in transcript expressions of Egr-1 (**i**), Integrin alpha 1, ITGA1 **(ii)**, and Crystallin alpha b, CRYAB (**iii**), in GDNF-GFRα1a-RET9 (**A**), GDNF-GFRα1a-RET51 (**B**), Forskolin (**C**), Y27632 (**D**) treated samples relative to that of control were normalized by geometric mean of top 2 reference genes in each subgroup; geometric mean of RPL19/RPL29; ACTB or GAPDH. Normalization by ACTB resulted in the over-estimation of target gene expression. Normalization by GAPDH led to either under- or over-estimation of target gene expression. Dotted line represents the 2-fold difference between treatment and control subjects, a cut off commonly used to distinguish significant changes from insignificant ones. Significant differences between fold changes normalized by various reference gene(s) were calculated using the paired Student’s *t test.* A value of p<0.05 was considered significant (**p<0.01; *p< 0.05)


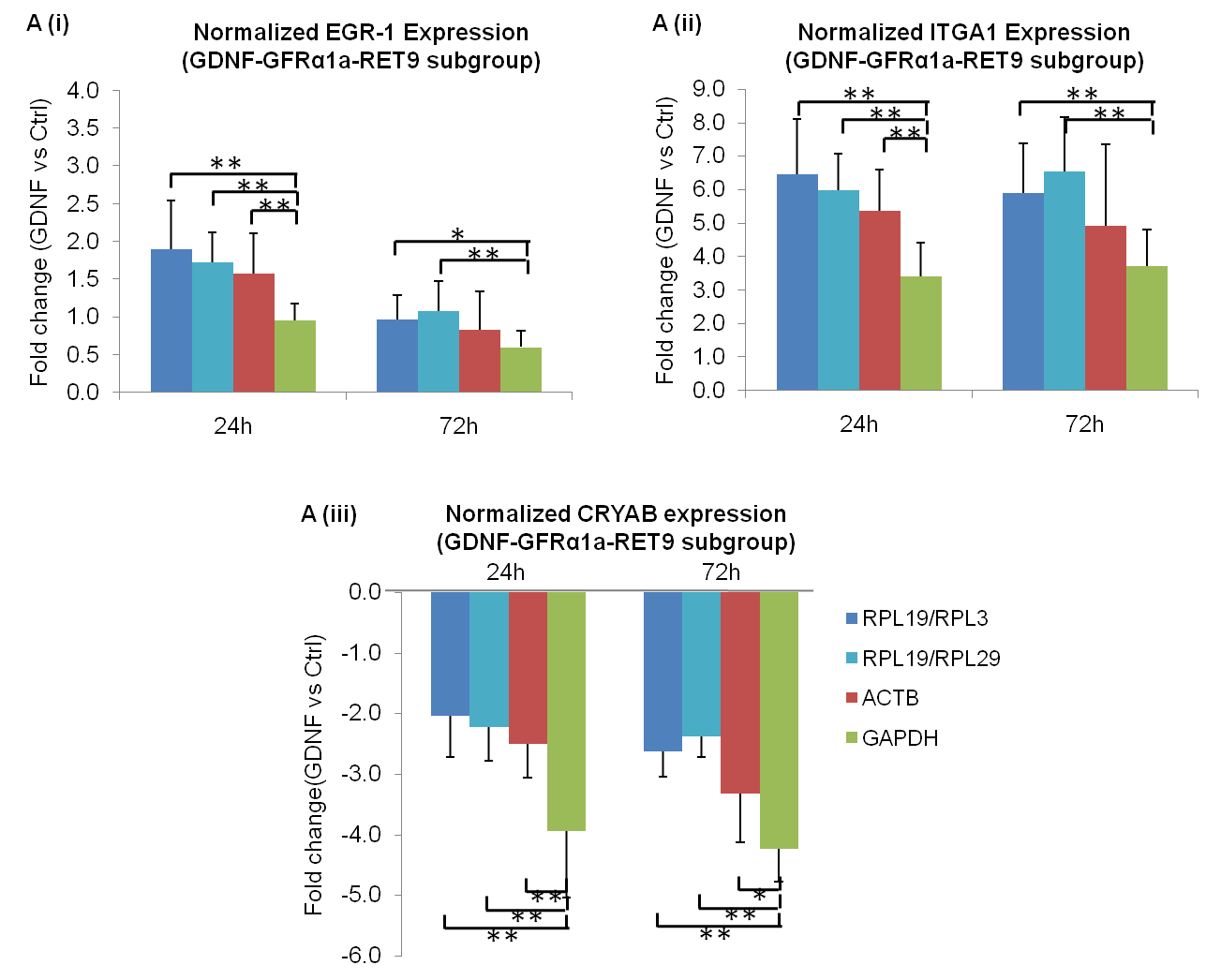


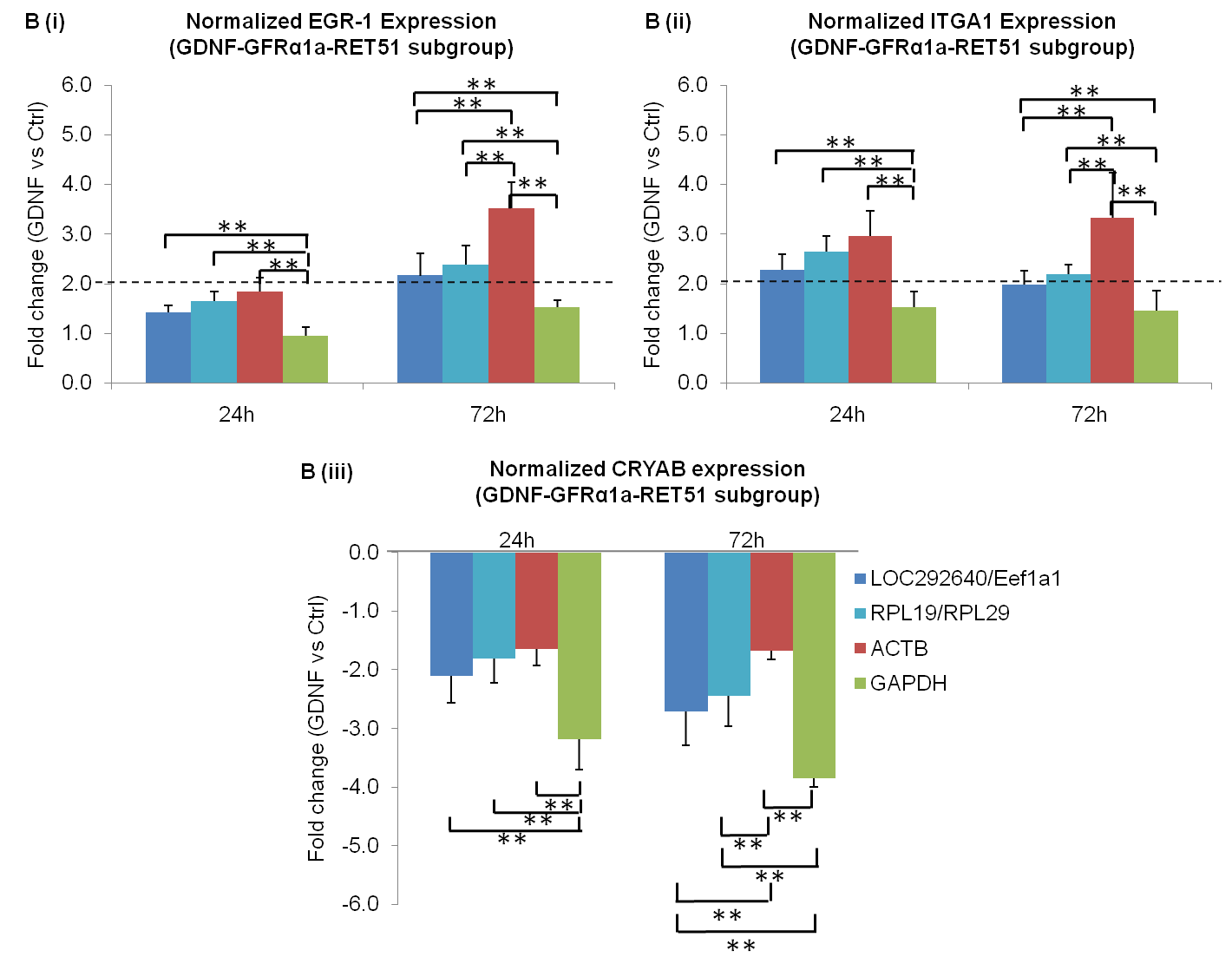


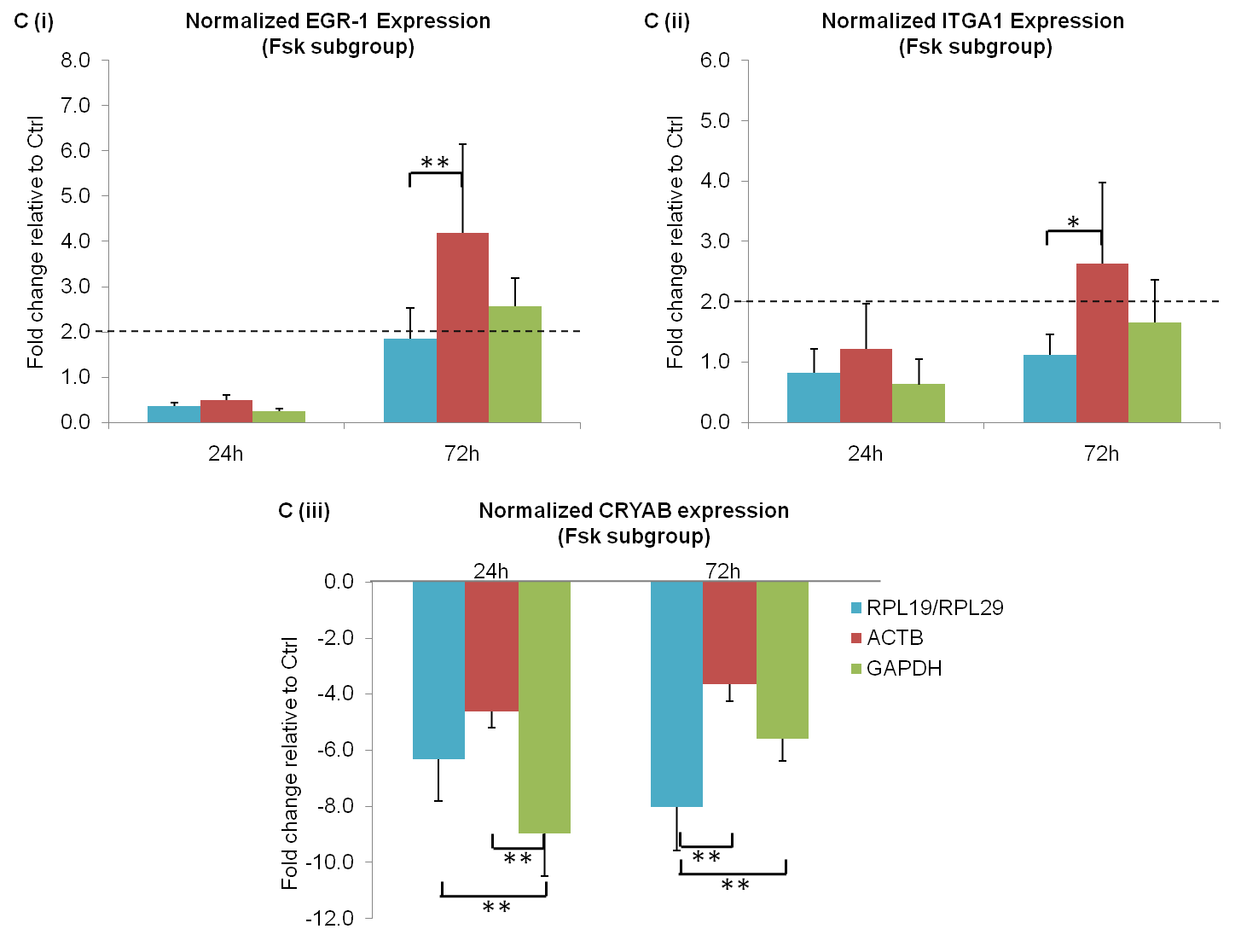


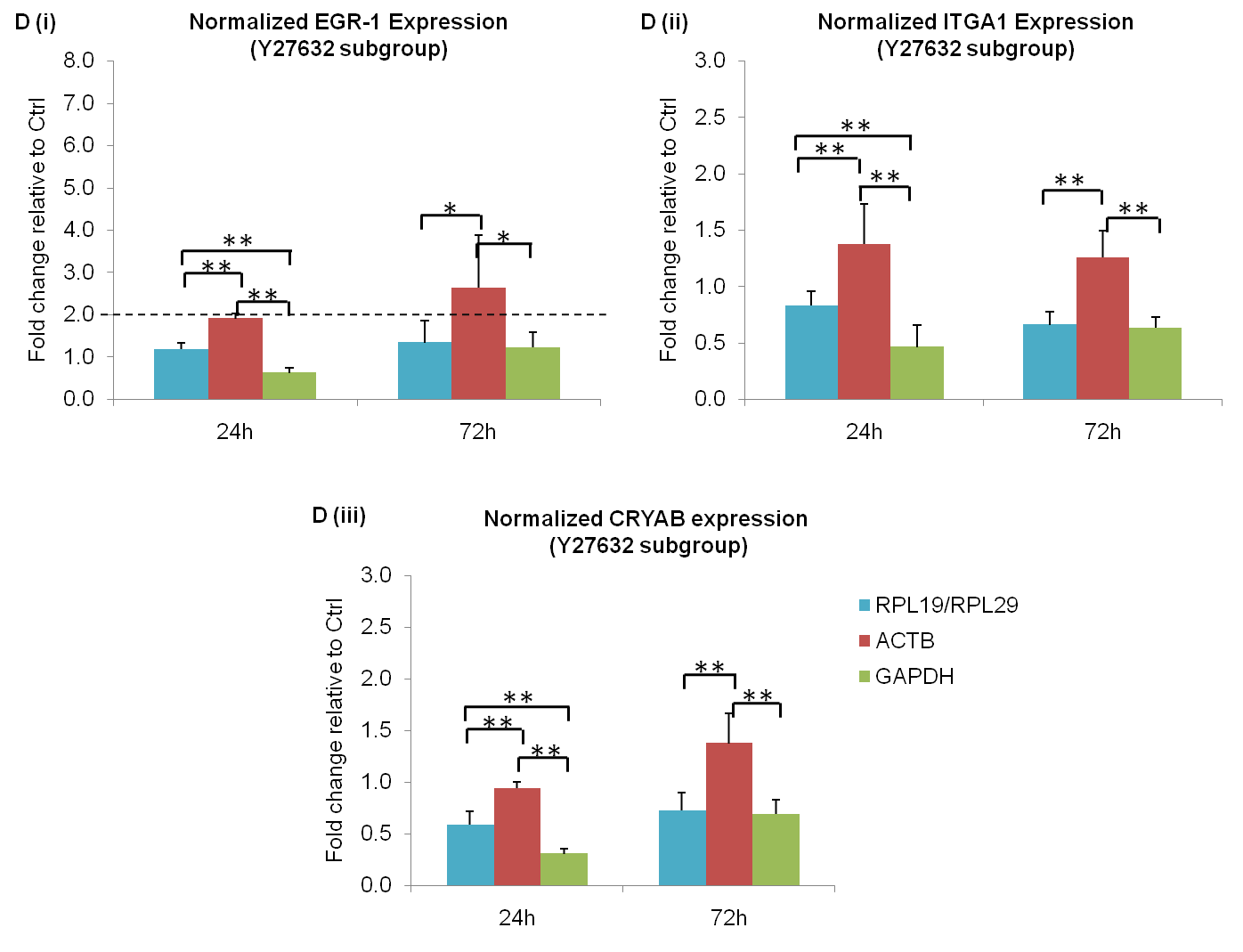

Supplement: Additional file 5 — Normalized target gene expression regulation in PC12 cells differentiated with GDNF, Forskolin and Y27632. Fold changes in transcript expressions of Egr-1 (i), Integrin alpha 1, ITGA1 (ii), and Crystallin alpha b, CRYAB (iii), in GDNF-GFRa1a-RET9 (A), GDNF-GFRa1a-RET51 (B), Forskolin (C), Y27632 (D) treated samples relative to that of control were normalized by geometric mean of top 2 reference genes in each subgroup; geometric mean of RPL19/RPL29; ACTB or GAPDH. Normalization by ACTB resulted in the over-estimation of target gene expression. Normalization by GAPDH led to either under- or over-estimation of target gene expression. Dotted line represents the 2-fold difference between treatment and control subjects, a cut off commonly used to distinguish significant changes from insignificant ones. Significant differences between fold changes normalized by various reference gene(s) were calculated using the paired Student's t test. A value of p < 0.05 was considered significant (**p < 0.01; *p < 0.05) [file 1471-2164-11-75-S5.DOCX]
